# Supplementary material for: Targeting Hedgehog Pathway and DNA Methyltransferases in Uterine Leiomyosarcoma Cells
Source: Cells. 2020 Dec 31;10(1):53. doi: 10.3390/cells10010053 (PMC7824187; doi:10.3390/cells10010053)

Table S1: qRT-PCR primers sequences

Gene / Symbol	Forward sequence	Reverse sequence	Amplicon Size (pb)	Melt temperature (°C)
SHH	TGCTGCTAGTCCTCGTCTCCT	TTTTGGGGTGCCTCCTCTT	90	F: 62.41<br>R: 58.72
DHH	TGATGACCGAGCGTTGTAAG	GCCAGCAACCCATACTTGT	196	F: 58.01<br>R: 58.04
IHH	CATTGAGACTTGACTGGGCAAC	AGAGCAGGCTGAGTTGGGAGTCGC	152	F: 59.77<br>R: 68.53
PTCH1	GAGCAGATTTCCAAGGGGAAGG	ATGAGGAGGCCCACAACCAA	137	F: 60.95<br>R:61.43
SMO	TGAAGGCTGCACGAATGAGG	CTTGGGGTTGTCTGTCCGAA	88	F: 60.67<br>R: 59.89
GLI1	AGCCTTCAGCAATGCCAGTGAC	GTCAGGACCATGCACTGTCTTG	151	F: 63.67<br>R: 61.45
GLI2	CTGTGGGTTAGGGATGGACTG	GTAAAGTGGGTGGACGTTGCA	147	F: 59.79<br>R: 61.08
GLI3	GTGCTCCACTCGAACAGA	TCCAGGACTTTCATCCTCATTAGA	76	F: 56.61<br>R: 58.72
DNMT1	CCATCAGGCATTCTACCA	CGTTCTCCTTGTCTTCTCT	132	F: 53.52<br>R: 53.85
DNMT3a	TATTGATGAGCGCACAAGAGAGC	GGGTGTTCCAGGGTAACATTGAG	111	F: 61.29<br>R: 61.12
DNMT3b	GAGTCCATTGCTGTTGGAACCG	ATGTCCCTCTTGTCGCCAACCT	305	F: 62.02<br>R: 63.94
B2M	CAGCCCAAGATAGTTAAGTG	CCCTCCTAGAGCTACCTGT	262	F: 53.40<br>R: 56.75

Table S2: Description of the antibodies used for WB analyses

Antibodies	Brand	Species Raised, Monoclonal or Polyclonal	Dilution
SMO	GeneTex	Rabbit, polyclonal	1:500
GLI1	Cell Signaling	Rabbit, monoclonal	1:1000
GLI2	BioRad	Rabbit, monoclonal	1:1000
GLI3	GeneTex	Rabbit, polyclonal	1:1000
DNMT1	Santa Cruz	Rabbit, polyclonal	1:1000
DNMT3a	Santa Cruz	Goat, polyclonal	1:1000
PCNA	Santa Cruz	Rabbit, polyclonal	1:1000
PARP	Santa Cruz	Rabbit, polyclonal	1:1000
RHOGDI	Santa Cruz	Rabbit, polyclonal	1:1000
β-ACTIN	Sigma Aldrich	Mouse, monoclonal	1:5000

Figure S3

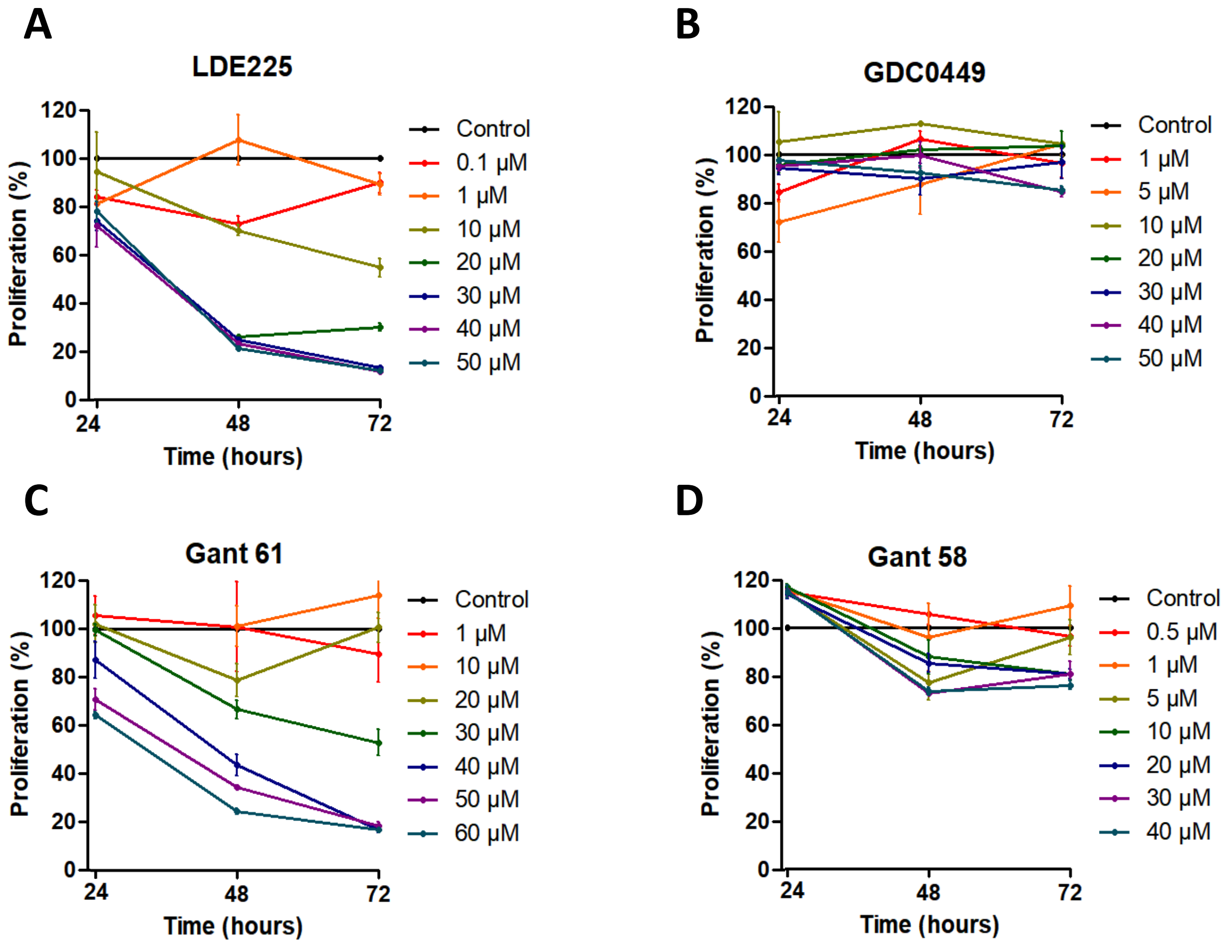

A
LDE225
Proliferation (%)
Time (hours)
Control
0.1 μM
1 μM
10 μM
20 μM
30 μM
40 μM
50 μM
B
GDC0449
Proliferation (%)
Time (hours)
Control
1 μM
5 μM
10 μM
20 μM
30 μM
40 μM
50 μM
C
Gant 61
Proliferation (%)
Time (hours)
Control
1 μM
10 μM
20 μM
30 μM
40 μM
50 μM
60 μM
D
Gant 58
Proliferation (%)
Time (hours)
Control
0.5 μM
1 μM
5 μM
10 μM
20 μM
30 μM
40 μM

Figure S4

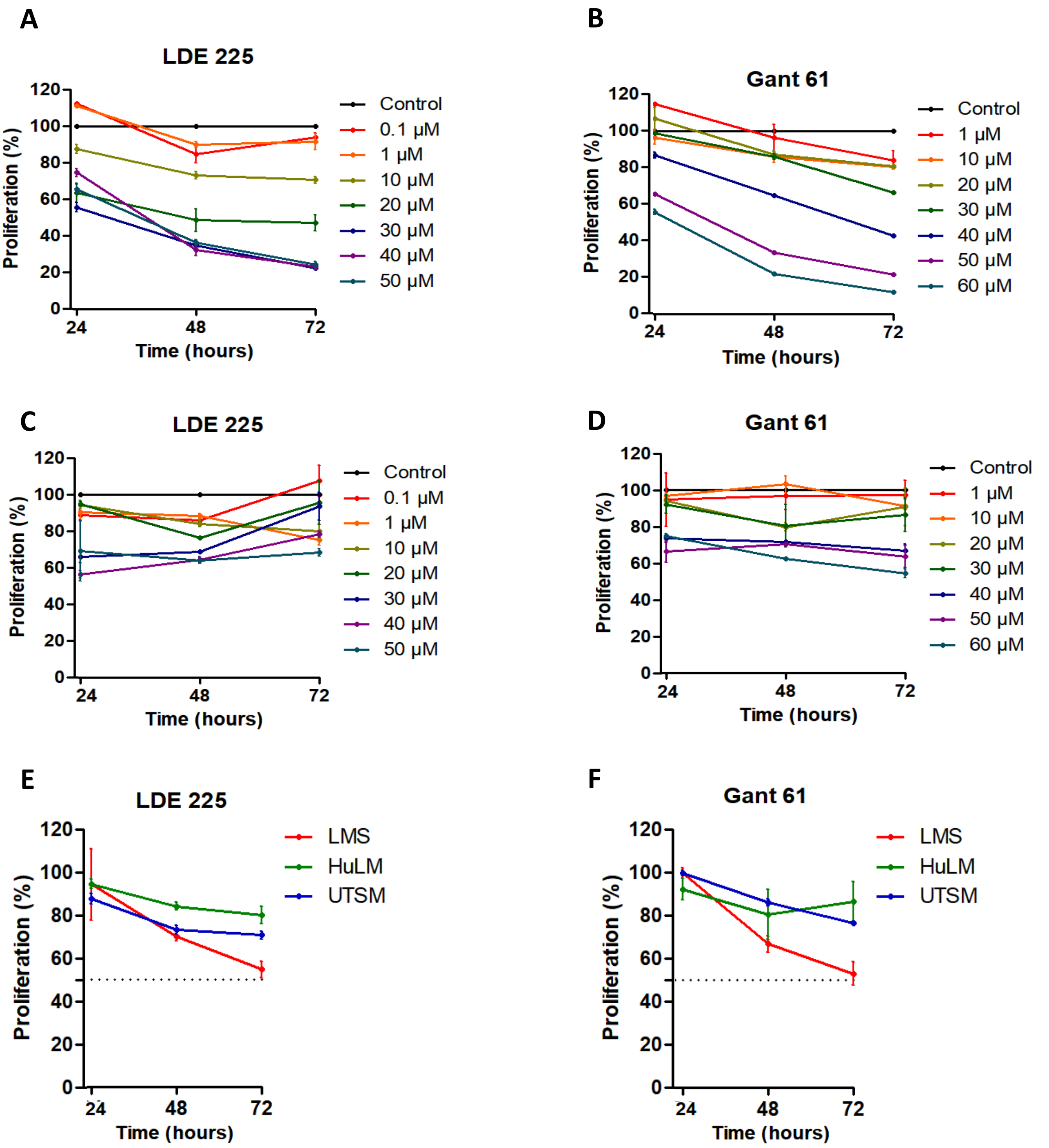
A
LDE 225
Proliferation (%)
Time (hours)
Control
0.1 µM
1 µM
10 µM
20 µM
30 µM
40 µM
50 µM
B
Gant 61
Proliferation (%)
Time (hours)
Control
1 µM
10 µM
20 µM
30 µM
40 µM
50 µM
60 µM
C
LDE 225
Proliferation (%)
Time (hours)
Control
0.1 µM
1 µM
10 µM
20 µM
30 µM
40 µM
50 µM
D
Gant 61
Proliferation (%)
Time (hours)
Control
1 µM
10 µM
20 µM
30 µM
40 µM
50 µM
60 µM
E
LDE 225
Proliferation (%)
Time (hours)
LMS
HuLM
UTSM
F
Gant 61
Proliferation (%)
Time (hours)
LMS
HuLM
UTSM

Figure S5

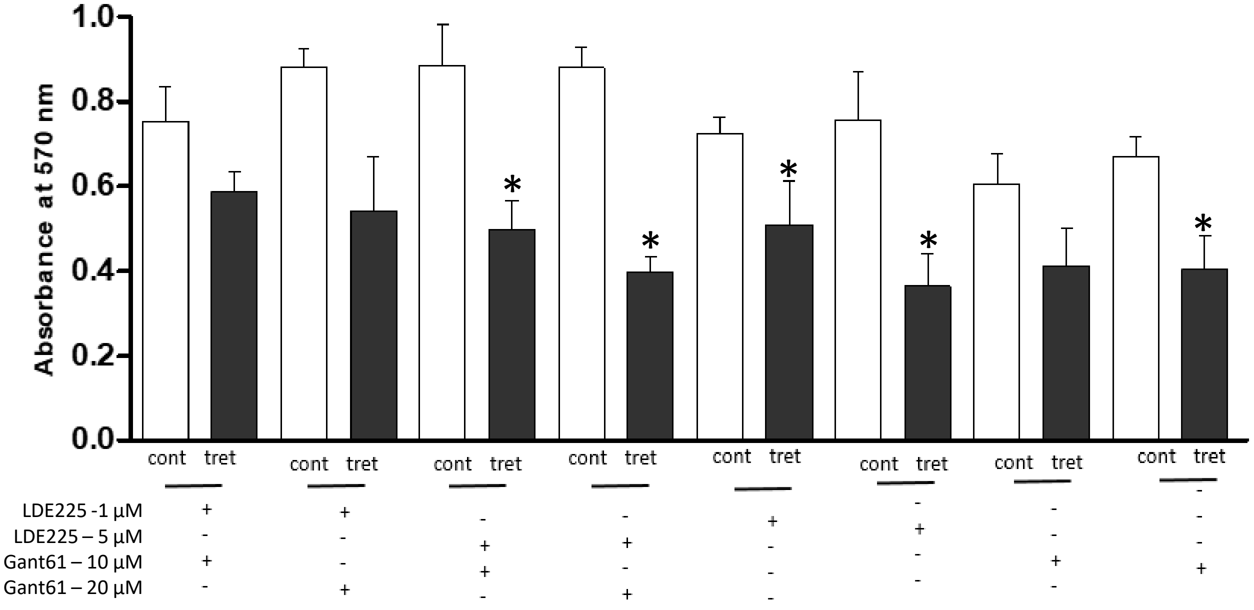
Absorbance at 570 nm
1.0
0.8
0.6
0.4
0.2
0.0
cont tret
*
LDE225 -1 µM
LDE225 – 5 µM
Gant61 – 10 µM
Gant61 – 20 µM
+
-

Figure S6

A

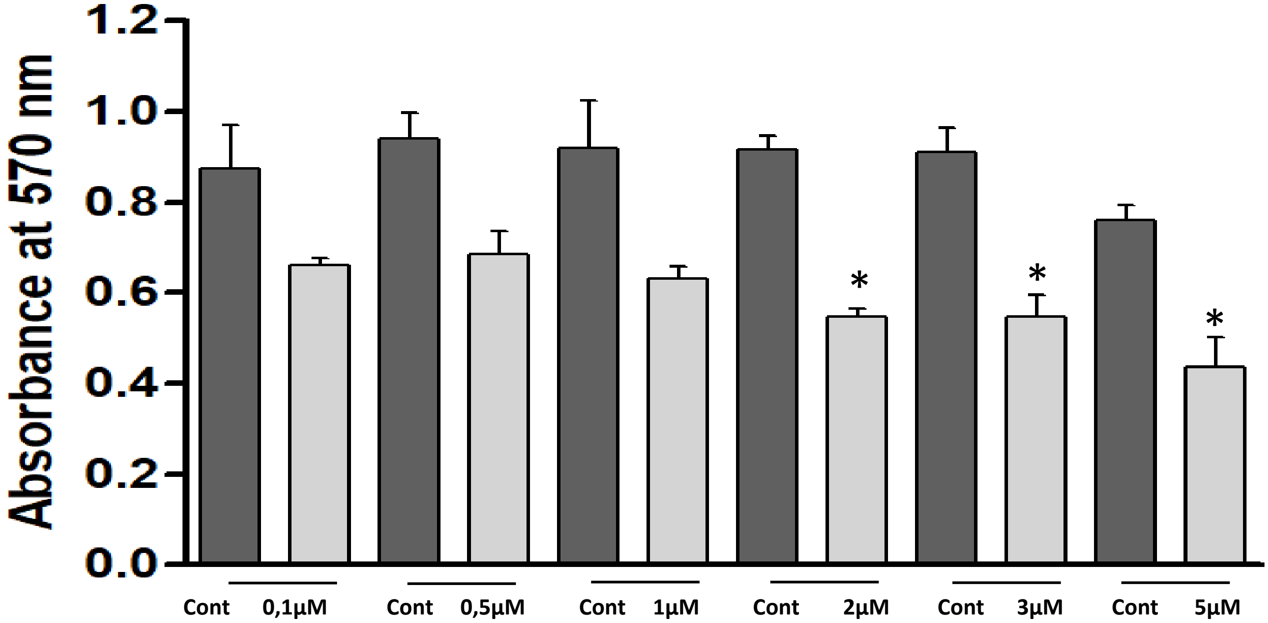

Absorbance at 570 nm
1.2
1.0
0.8
0.6
0.4
0.2
0.0
*
*
*
Cont
0,1µM
Cont
0,5µM
Cont
1µM
Cont
2µM
Cont
3µM
Cont
5µM

B

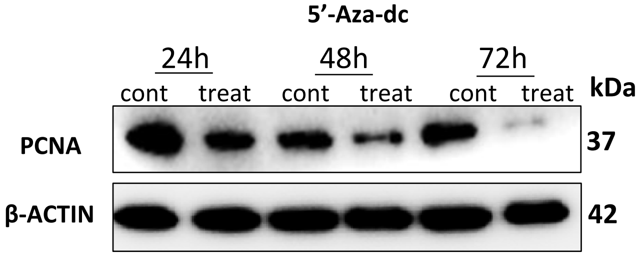

5'-Aza-dc
24h
48h
72h
cont
treat
cont
treat
cont
treat
kDa
PCNA
37
β-ACTIN
42

Figure S7

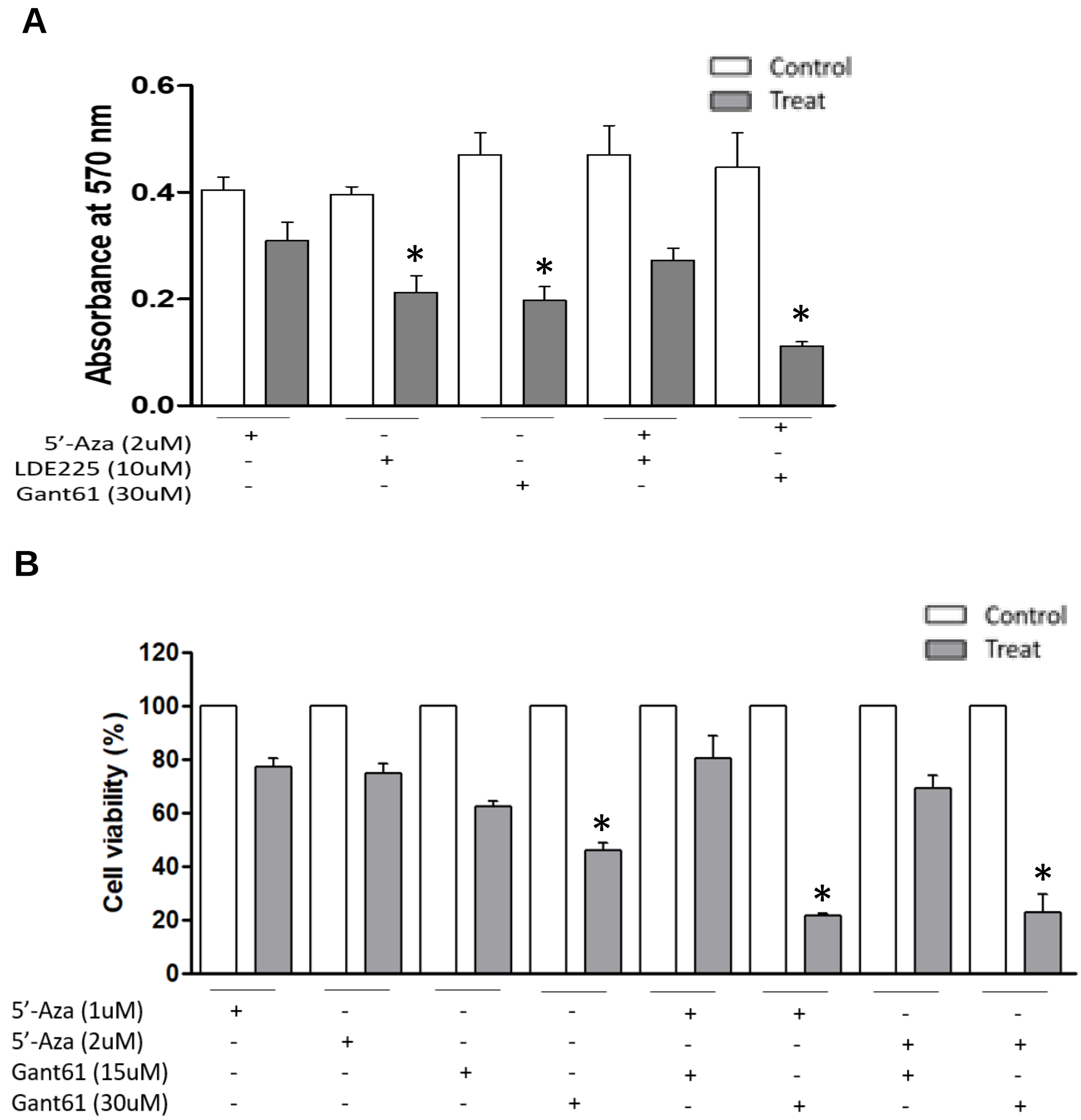
A
Control
Treat
0.6
0.4
0.2
0.0
Absorbance at 570 nm
*
*
*
5'-Aza (2uM) + - - + +
LDE225 (10uM) - + - + -
Gant61 (30uM) - - + - +
B
Control
Treat
120
100
80
60
40
20
0
Cell viability (%)
*
*
*
5'-Aza (1uM) + - - - + + - -
5'-Aza (2uM) - + - - - - + +
Gant61 (15uM) - - + - + - + -
Gant61 (30uM) - - - + - + - +

Figure S8

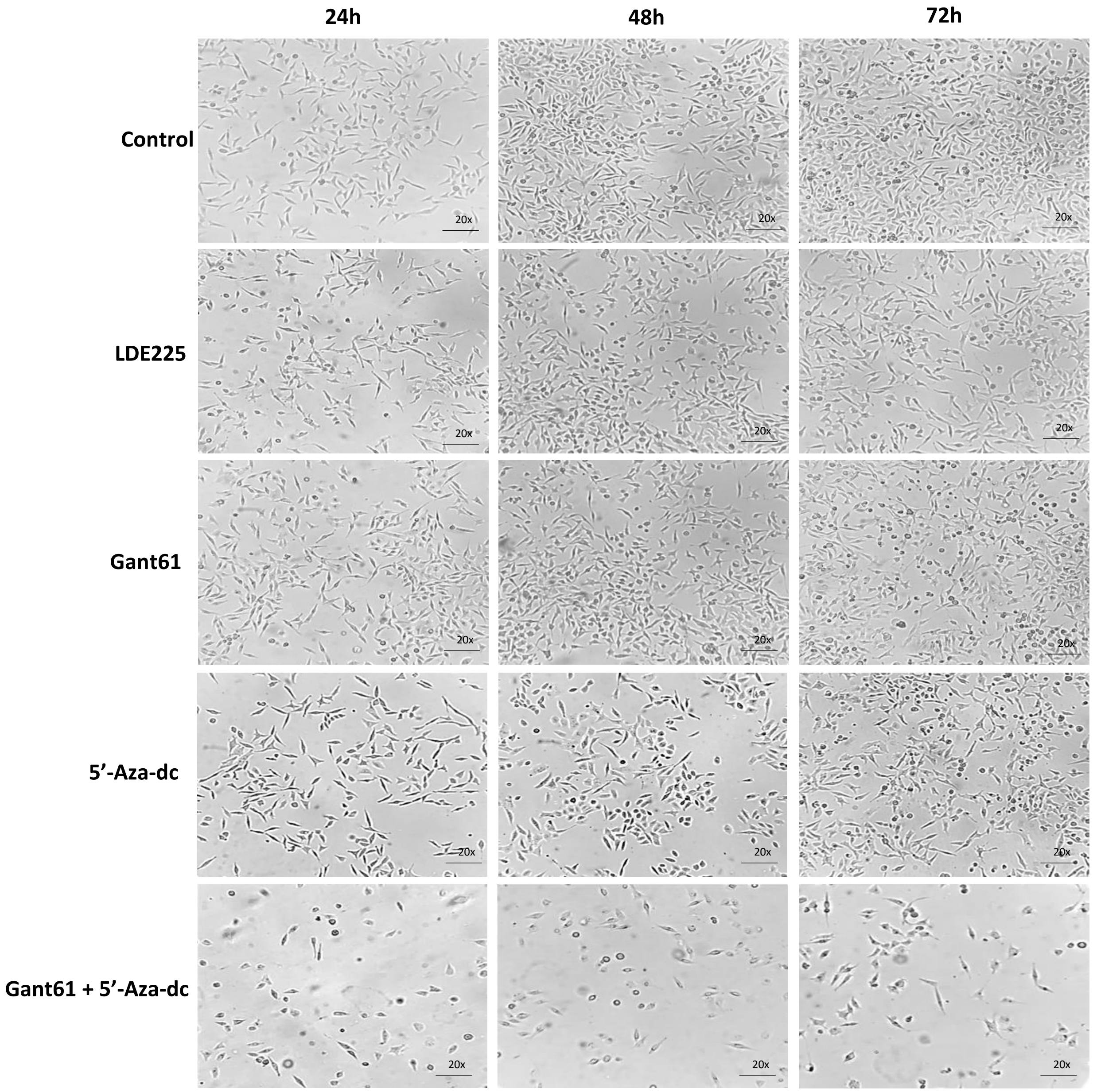

Supplement: Supplementary file 1 [file cells-10-00053-s001.pdf]
